# Overuse‐Induced Muscle Disorder: Establishing a Rat Model to Unravel the Role of Fibro‐Adipogenic Progenitor Cells in Intramuscular Fibrosis

**DOI:** 10.1002/jor.70076

**Published:** 2025-09-30

**Authors:** Hiroyori Fusagawa, Tatsuya Sato, Azuma Naito, Nao Tokuda, Nao Yamauchi, Akiyoshi Uezumi, Madoka Uezumi, Yuki Saito, Minami Fusagawa, Hiroyuki Takashima, Nobutoshi Ichise, Toshifumi Ogawa, Takuro Karaushi, Noritsugu Tohse, Brian Feeley, Xuhui Liu, Atsushi Teramoto, Takashi Yamada

**Affiliations:** ^1^ San Francisco Veterans Affairs Health Care System San Francisco California USA; ^2^ Department of Orthopaedic Surgery University of California San Francisco California USA; ^3^ Division of Cellular Physiology and Signal Transduction, Department of Physiology Sapporo Medical University School of Medicine Sapporo Japan; ^4^ Department of Orthopaedic Surgery Sapporo Medical University School of Medicine Hokkaido Japan; ^5^ Division of Cardiovascular‐Kidney‐Metabolic Medicine, Department of Internal Medicine Sapporo Medical University School of Medicine Hokkaido Japan; ^6^ Graduate School of Health Sciences Sapporo Medical University Hokkaido Japan; ^7^ Division of Cell Heterogeneity, Medical Research Center for High Depth Omics, Medical Institute of Bioregulation Kyushu University Fukuoka Japan; ^8^ Department of Anatomy Sapporo Medical University School of Medicine Hokkaido Japan; ^9^ Department of Pathology Sapporo Medical University School of Medicine Hokkaido Japan; ^10^ Division of Biomedical Science and Engineering, Faculty of Health Sciences, Biomedical Science and Engineering Hokkaido University Hokkaido Japan; ^11^ Graduate School of Biomedical and Health Sciences Hiroshima University Hiroshima Japan

**Keywords:** fibrosis, isometric contraction, MSCs/FAPs, NMES, overuse

## Abstract

Overuse‐induced muscle disorders (OIMD) frequently occur in athletes due to excessive and improper use under high physical demand, often leading to muscle pain and weakness. Limited studies have shown intramuscular fibrosis in OIMD, with fibro‐adipogenic progenitors (FAPs), also known as mesenchymal stromal cells (MSCs), playing a crucial role in this fibrosis. This study aimed to develop a rat OIMD model using neuromuscular electrical stimulation‐induced isometric exercise (NMES‐ISO) and to investigate mechanisms by which excessive exercise without adequate rest leads to OIMD. We hypothesize that daily NMES‐ISO would cause muscle weakness and fibrogenesis mediated by FAP activation triggered by muscle fiber damage. Male Wistar rats received daily NMES‐ISO loading on the plantar flexor muscles at a lengthened position. Two weeks of NMES‐ISO led to decreased muscle torque without muscle mass reduction. Masson‐Trichrome stain revealed collagen‐rich fibrogenic lesions in the gastrocnemius muscles, while Evans blue stain detected no muscle fiber damage during the first 5 days. MRI showed increased T2*wi signals correlating with fibrogenic areas. Excessive NMES‐ISO stimulated FAP proliferation. This study established a rat model of OIMD using NMES‐ISO, characterized by muscle weakness and intramuscular fibrogenesis. Contrary to our hypothesis, FAP activation occurred without overt muscle injury, suggesting excessive mechanical loading may directly trigger FAP proliferation and fibrogenesis. It was cautioned that even relatively safe isometric contraction training could lead to FAP activation and intramuscular fibrosis without proper methods and recovery periods.

## Summary

- Our study demonstrated a novel overuse‐induced muscle disorder model in rats using neuromuscular electrical stimulation, enabling investigation of the mechanisms by which continuous loading leads to muscle dysfunction.
- This model activated mesenchymal stromal cells without evident muscle injury and caused intramuscular fibrosis, challenging the traditional concept that repetitive muscle injury underlies overuse.
- These findings provide a new perspective on the pathogenesis and treatment of overuse‐induced muscle disorders.

## Graphical abstract

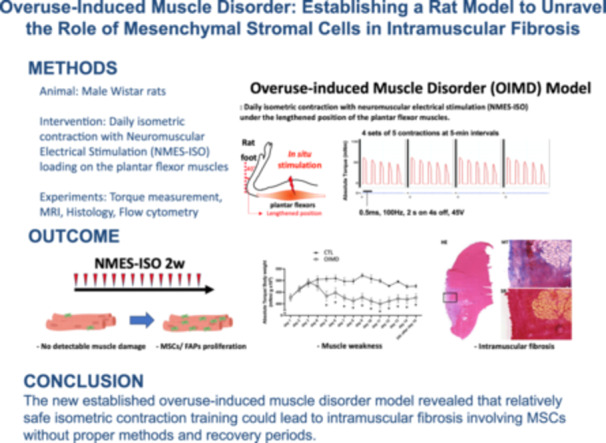

## 1 Introduction

Overuse‐induced muscle disorder (OIMD) occurs in athletes who train excessively, as well as in physically demanding laborers who do not get sufficient rest [1, 2, 3]. Patients with tendonitis, considered an overuse syndrome, often report symptoms associated with OIMD, such as persistent muscle pain and muscle weakness, highlighting the need for a muscle‐focused therapeutic approach [4]. However, the difficulty in obtaining affected muscle samples from highly active patients with OIMD has justifiably hindered progress in this area of research. Studies using animal models have revealed that OIMD is affected by intramuscular fibrosis resulting from prolonged chronic inflammation [1, 5]. This model effectively induced OIMD by creating conditions of muscle overuse and improper use through daily food intake and activities that forced reaching maneuver within narrow tunnels. However, the mechanism by which repetitive motion stress causes muscle disorders characterized by intramuscular fibrosis has not been fully elucidated. Furthermore, this type of model makes it difficult to precisely set the number of repetitions for exercise and evaluate specific types of muscle contractions, and it requires unique equipment devised at the facility.

Mesenchymal stromal cells (MSCs), also known as mesenchymal progenitors (MPs) and fibro/adipogenic progenitors (FAPs), are non‐myogenic cells located in the interstitial space between muscle fibers [6]. These names all refer to the same multipotent cells within muscles that have stem cell antigen‐1 (Sca‐1) and platelet‐derived growth factor receptor alpha (PDGFRα) as key surface markers [7]. Recently, these cells have been found to differentiate not only into fibroblasts, adipocytes, and osteocytes, thus MSCs would most accurately describe their characteristics [8]. However, FAPs are conventionally used more broadly; therefore, this study will use the term FAPs for consistency. Other mesenchymal stem cell populations are believed to reside in muscle including pericytes and bone marrow‐derived stem cells. This study is focused on FAPs, rather than all such stem cell subpopulations broadly. In recent years, it has been suggested that FAPs play a crucial role in intramuscular fibrosis [8], and the involvement of FAPs has been increasingly recognized in various diseases [9, 10, 11, 12].

Neuromuscular electrical stimulation (NMES) induces muscle contraction by stimulating both the innervating nerve of the target muscle and the muscle itself through surface electrodes attached to the body. Unlike voluntary exercise, NMES allows the recruitment of all muscle fibers by adjusting the stimulus intensity, thereby ensuring that each subject receives an equivalent exercise load. Due to this characteristic, NMES allows for highly reproducible exercise loading, which can be finely turned by controlling the number of repetitions. This enables a more precise investigation of exercise frequency‐dependent muscle pathology. Additionally, exercise generally involves a combination of three major contraction modalities, namely isometric contraction (ISO), concentric contraction, and eccentric contraction (ECC) [13], all of which can be independently reproduced by NMES, thereby allowing for the evaluation of their individual effects.

Although ISO is considered relatively safe and is a preferred exercise protocol in sports and rehabilitation, it is known to cause damage at the myofibrillar level [14]. Furthermore, there is evidence for greater damage when the muscle works at longer compared with shorter lengths [15]. Previously, we established exercise‐mimic protocols using NMES‐induced ISO (NMES‐ISO) to apply loadings to various rodent muscles under anesthesia, demonstrating beneficial effects on muscle hypertrophy and function improvement with alternate‐day loading [16, 17, 18].

In this study, we aimed to develop a rat model of OIMD using NMES to investigate how repeated muscle contraction loading leads to muscle weakness and intramuscular fibrosis, focusing on muscle fiber damage and the activation of FAPs through a temporal evaluation approach. We hypothesize that daily NMES‐ISO loading without rest days in a muscle lengthening position induces muscle damage with prolonged force depression, leading to intramuscular fibrosis. We also hypothesize this process is mediated by FAP activation triggered by muscle fiber damage.

## 2 Methods

### 2.1 Experimental Approval and a Rat Model for Overuse‐Induced Muscle Disorder (OIMD)

All experimental protocols were reviewed and approved by the Ethics Committee on Animal Experiments of Sapporo Medical University (No. 23‐082, Sapporo, Japan). Animal care was performed in strict accordance with institutional guidelines. Male Wistar rats (6–7 weeks old, *n* = 25) were supplied by Sankyo Labo Service (Sapporo, Japan). The rats were housed in an environmentally controlled room (24 ± 2°C, 12 h: 12 h light–dark cycle) and given food and water ad libitum.

### 2.2 Experimental Design

To elucidate the mechanism of OIMD, we performed three separate experiments.

#### 2.2.1 Experiment 1

We first created an OIMD model in rat plantar flexor muscles using isometric contraction (ISO) with NMES (NMES‐ISO) and assessed their physiological phenotype. Male Wistar rats (6 weeks old, *n* = 10) were assigned to three groups: ISO 2w group (ISO 2w, *n* = 6), ISO 2w + sedentary 2w group (ISO 2w + SED 2w, *n* = 2), and ISO 2w + sedentary 4w group (ISO 2w + SED 4w, *n* = 2). The ISO 2w group was subjected to 2 weeks of daily NMES‐ISO loading on the left leg, with the right leg serving as the control. Twenty‐four hours after the last NMES‐loading session, the rats were killed by cervical dislocation under isoflurane anesthesia. In vivo plantar flexor torque was measured in the plantar flexor muscles over time during the observation period. The ISO 2w + SED 2w group was subjected to 2 weeks of ISO loading on both legs, followed by a 2‐week sedentary period of no loading. The ISO 2w + SED 4w group was subjected to 2 weeks of ISO loading on both legs, followed by a 4‐week sedentary period of no loading. MRI imaging of the lower leg was performed periodically throughout the observation period. The gastrocnemius muscles were dissected from each animal and subjected to histological analysis.

**Figure 1:**
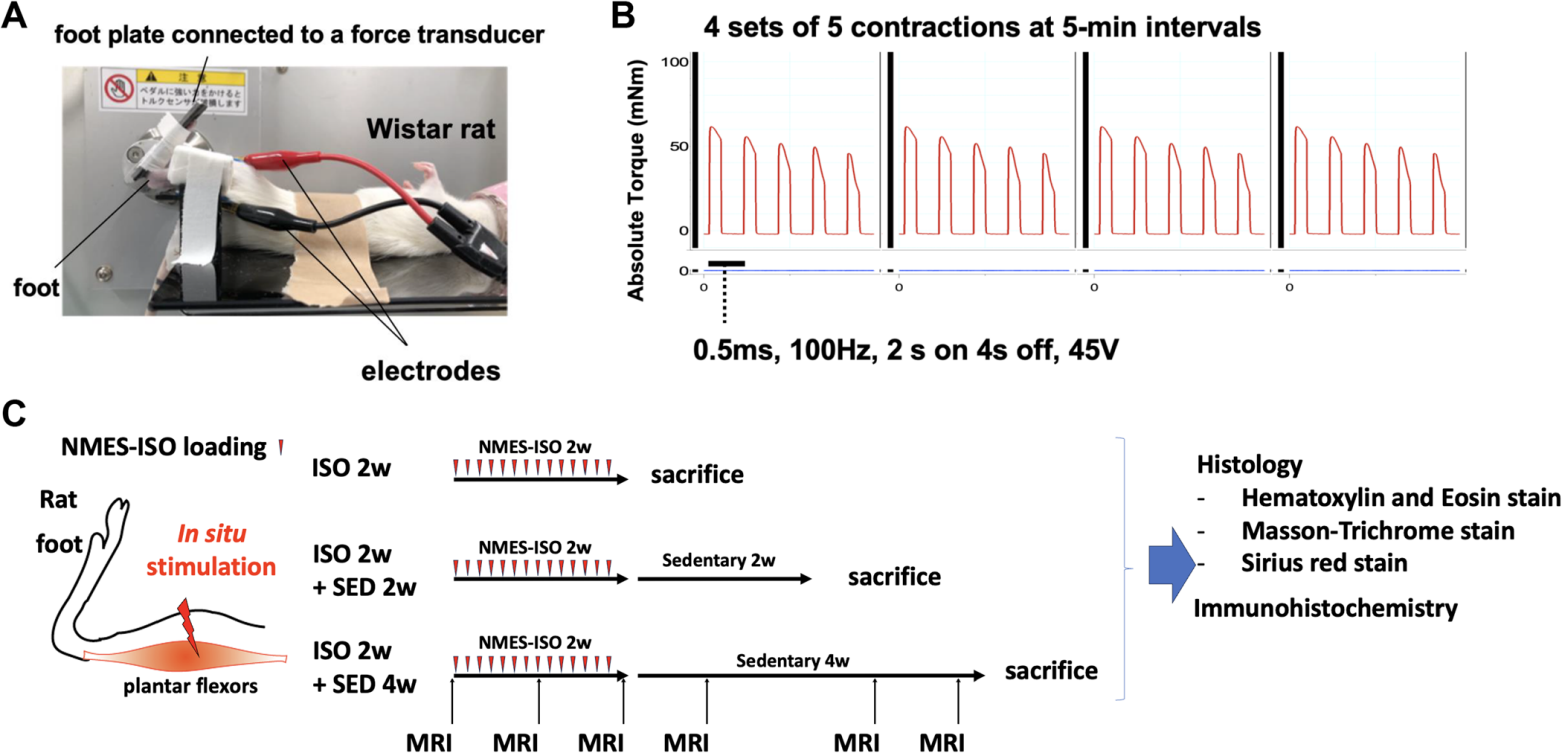
(A) Representative image of the neuromuscular electrical stimulation (NMES) setup. (B) Typical torque traces of NMES‐induced isometric contraction exercise (ISO) (4 sets of 5 contractions at 5‐min intervals consisting of 2‐s contraction with 100 Hz, 0.5 ms, 45 V every 6 s). (C) Schematic overview of the Experiment 1.

Throughout the NMES‐ISO loading sessions, rats were anesthetized by isoflurane inhalation. Rats were placed supine on a platform, and their foot was secured in a foot plate connected to a torque sensor (S‐14154; Takei Scientific Instruments) at an angle of 40° dorsiflexion (i.e., 50° relative to the tibia), a position in which the plantar flexor muscles, including the gastrocnemius, plantaris, and soleus muscles, are in a lengthened state. Plantar flexor muscles were stimulated supramaximally (45 V) using a pair of surface electrodes (BlueSensor, Ambu, surface area of 0.785 cm^2^) that were placed on the skin and strapped by tape to the posterior surface of the calf (Figure 1A). Stimulation parameters were same as those described by Ashida et al. [16]: 0.5‐ms monophasic rectangular pulse, 2‐s contraction every 6 s. Each session consisted of four sets of five contractions at 5‐min intervals and was carried out every day for 2 weeks. The torque production was measured during NMES (Figure 1B). An overview of Experiment 1 is shown in Figure 1C.

#### 2.2.2 Experiment 2

To investigate the time course of muscle fiber damage, 6‐week‐old male Wistar rats (*n* = 5) were divided into five groups (ISO 1 day, ISO 2 days, ISO 3 days, ISO 4 days, ISO 5 days). Each group (*n* = 1) was killed for histology after NMES‐ISO loading for 1–5 consecutive days.

#### 2.2.3 Experiment 3

To investigate the involvement of interstitial cells, 6‐week‐old male Wistar rats (*n* = 10) were divided into four groups: CTL, ISO 3 days, ISO 4 days, and ISO 5 days. The ISO 3 days group (*n* = 3) and ISO 5‐days group (*n* = 3) were subjected to NMES‐ISO loading on both legs for 3 and 5 days, respectively, and then killed for histological analysis. The ISO 4‐day group (*n* = 2) was subjected to ISO loading on both legs for 4 days and then killed along with the unloaded CTL group (*n* = 2) for fluorescence‐activated cell sorting (FACS) analysis.

Changes in muscle weight and torque production were compared with the contralateral side, which served as the control (CTL). On the other hand, in other experiments, the CTL used was the muscle from rats that did not undergo NMES‐ISO on either side.

### 2.3 In Vivo Torque Measurement With Electrical Stimulation

The torques were recorded on a computer and analyzed using LabChart version 8 before loading NMES‐ISO protocol at the same setting as those in set of NMES‐ISO loading except the foot angles remaining neutral 0° plantarflexion position. The maximum in vivo torque was measured at a stimulation frequency of 100 Hz (duration 600 ms) with short (0.5 ms) current pulses such that only one action potential was triggered by each pulse as previously described [16]. Normalized torque was calculated as the ratio of in vivo absolute torque to the whole body weight.

### 2.4 MRI

We performed in vivo ^1^H‐MRI studies using a 7‐Tesla MRI spectrometer (Pharmascan 70/16; Bruker) with a quadrature transceiver volume coil of 72 mm in inner diameter in Experiment 1, as previously described [19] with some arrangements. Each rat (*n* = 6) was positioned in a bed, which allowed delivery of anesthesia. Respiratory frequency was monitored with a pressure probe and kept between 60 and 90 breaths/min. Low‐resolution After setting the position to capture, T2‐weighted fast spin‐echo images (T2wi) were acquired in axial, sagittal and coronal planes with the following experimental parameters: TEeff = 30 ms; TR = 3 s; ETL = 6; FOV = 4 × 4 cm^2^; MTX = 256 × 256; number of signal average (NSA) = 1—for axial, coronal, and sagittal sections; slice thickness/gap = 1.2/0.5 mm, 1.0/0.5 mm, and 1.0/0.5 mm; respectively,; experimental time = 2 min 33 s, 1 min 36 s, and 2 min 6 s, respectively. Similarly, T2 * weighted images (T2*wi) were acquired in axial planes; TE = 3.34 ms; TR = 500 ms; flip angle = 50; FOV = 4 × 4 cm^2^; MTX = 256 × 256; NSA = 1. MRI data were acquired and processed on a Linux computer using Paravision 5.1 software (Bruker BioSpin GmbH).

### 2.5 Histopathology

For histological analysis, the 1/3 proximal side of the medical gastrocnemius muscle was frozen in precooled isopentane and stored at −80°C until the following steps. In Experiment 2, for Evans blue dye (EBD) quantification, 1% (wt/vol) EBD solution (1 mg/10 g body wt) was intraperitoneally injected 8 h before sacrifice. Cryostat sections (10 µm in thickness) were stained with hematoxylin and eosin (HE). Fibrosis evaluation was carried out using a Masson trichrome staining kit (TRM‐1‐IFU, ScyTek Laboratories) and Sirius red stain kit (PSR‐IFU, ScyTek Laboratories) per the manufacturer's instructions. For wheat germ agglutinin (WGA) staining, cryostat sections of the muscles were fixed in acetone/methanol for 15 min and then incubated in AF488‐conjugated WGA (W11261, Invitrogen) for 30 min at room temperature. For immunofluorescence, sections were fixed with 4% paraformaldehyde for 10 min followed by three 5‐min washes with 0.1% Tween in phosphate‐buffered saline (PBS). The sections were blocked with 1% bovine serum albumin in PBS for 1 h at room temperature and then incubated with primary antibodies against anti‐PDGFR⍺ (1:20; made in Goat; RSD Cat No. AF1062) overnight at 4°C. Sections were again washed with 0.1% Tween in PBS three times for 5 min each wash and then incubated with secondary antibodies: donkey anti‐goat Alexa Fluor 568 (1:400; Invitrogen Cat No. A11057). After the slides were washed three times with PBS for 5 min per wash, the slides were covered with mounting media with DAPI (VECTASHIELD, Vector Laboratories). Images were obtained from the serial sections with a BIOREVO BZ–X700 fluorescence microscope (Keyence) using 20× objective and DAPI, GFP, and TxRed filter sets. Binning was set to 2 × 2, the gain to 18 db and exposure times were 1/4000 s for DAPI, 1/2800 s for GFP, and 1/2800 s for TxRed channels. After staining, the percentage of the fibrotic area in Experiment 1, as well as the number of muscle fibers, cross‐sectional area (CSA), and whole muscle area in Experiment 2, were calculated using BZ‐X analyzer software (Keyence).

### 2.6 Isolation of Mesenchymal Stromal Cells

Medial and lateral gastrocnemius muscles were placed in PBS. Nerves, blood vessels, tendons, and fat tissues were carefully removed under a dissecting microscope. Trimmed muscles were minced and digested with 0.2% collagenase (Wako 034‐22363) for 30 min at 37°C. Digested muscles were passed through an 18‐gauge needle several times and further digested for 30 min at 37°C. Muscle slurries were filtered through a 100‐mm cell strainer (BD Biosciences) and then through a 40‐mm cell strainer (BD Biosciences). Erythrocytes were eliminated by treating the cells with Tris‐buffered 0.8% NH_4_Cl. Cells were then washed with 2.5% FBS in PBS and stained with Alexa Fluor 647 conjugated anti‐CD31 (1:120, Biorad MCA1334A647) and CD45 (1:300, Biolegend 202212), PE‐conjugated anti‐ PDGFR⍺ (1:300, R&D, FAB1062P), and biotinylated anti‐CD106 (VCAM‐1) (1:10, Miltenyi Biotec) for 30 min at 4°C. Cells were then stained with streptavidin‐Brilliant Violet 421 (1:300, Biolegend 405226) for 30 min at 4°C in the dark. Stained cells were counted using a FACS Aria II flow cytometer (BD Biosciences). Therefore, we excluded endothelial cells using CD31 and white blood cells using CD45, and collected PDGFRα‐positive FAPs based on the location of satellite cell populations identified by CD106 (VCAM‐1). All flow cytometry data were analyzed with FACSDiva and Flowjo software (BD Biosciences).

### 2.7 Statistics

Data are presented as mean ± SD. Data normality was examined with the Shapiro–Wilk test. Student's paired and unpaired *t*‐test were used to determine statistically significant differences as appropriate (see figure legends). When more than two groups were compared, one‐way ANOVA followed by Tukey's test or two‐way ANOVA followed by Sidak's test was used to analyze differences between groups. Kolmogorov–Smirnov test was used for comparison of frequency distributions about muscle fiber area. All statistics were performed on GraphPad Prism 8 and differences were considered statistically significant at *p* < 0.05 (*). A power analysis was performed using SigmaPlot (v.13, Systat Software Inc.), assuming changes in physiological measurements after ISO being 30% ± 20% of the CNT value [16]. With a power of 0.80 and an *α* of 0.05, this gives a sample size of four. Based on this, we used 4–6 animals in each group.

## 3 Results

### 3.1 Repeated Muscle Contraction Using NMES Results in Muscle Force Depression

**Figure 2:**
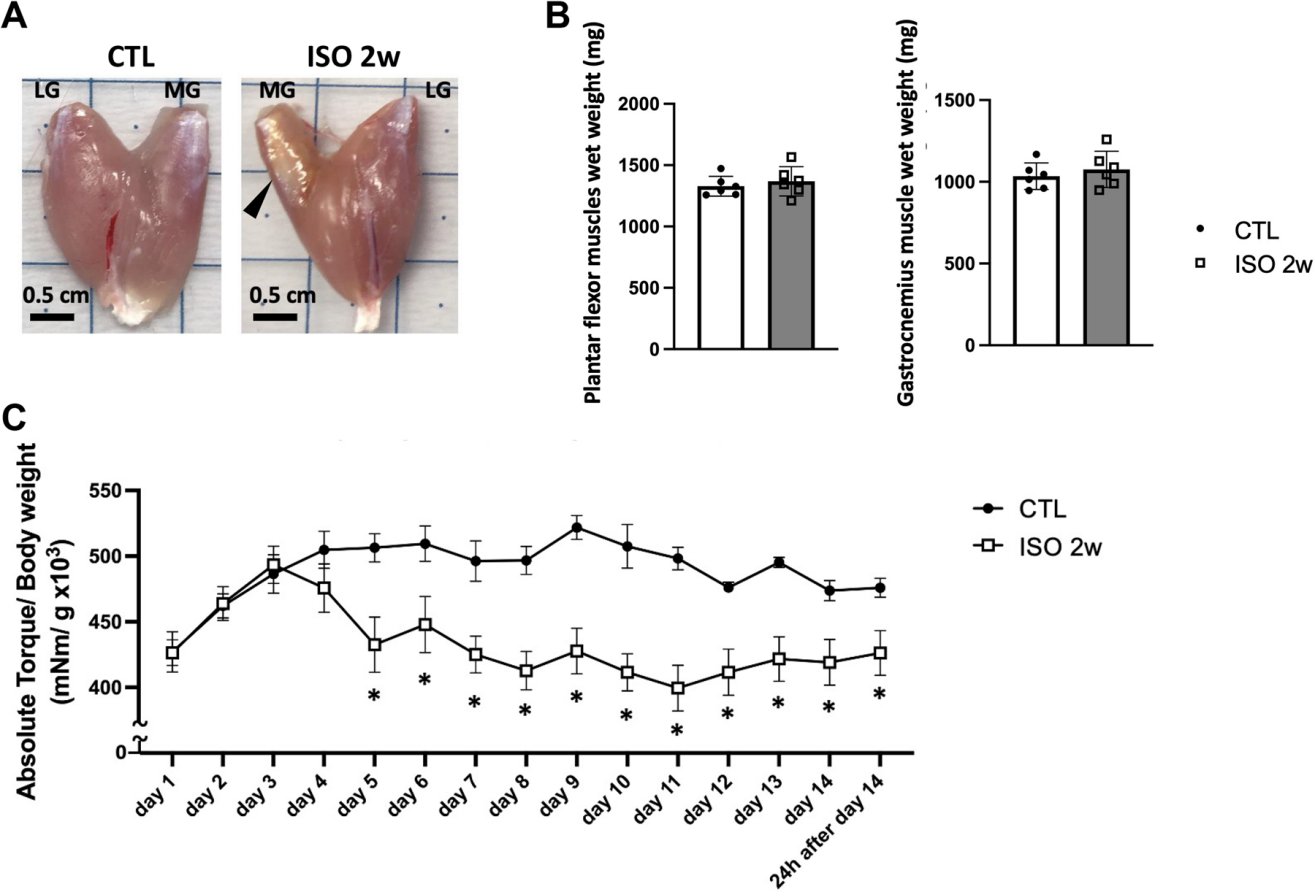
(A) Macroscopic dorsal view of the gastrocnemius muscle from the CTL side without exercise and the two‐week ISO side (ISO 2w). MG indicates the medial gastrocnemius muscle, and LG indicates the lateral gastrocnemius muscle. The arrowhead indicates connective tissue covering the proximal part of MG in the ISO 2w. (B) The wet weights of the plantar flexor muscles, including the soleus, plantaris, and gastrocnemius muscles, and the wet weights of the gastrocnemius muscle from the CTL legs (*n* = 6) and ISO 2w legs (*n* = 6). Paired *t*‐test was performed. *p < 0.05 CTL versus ISO 2w. (C) Mean (±SD) absolute torque normalized by body weight during two‐week ISO from the CTL side (*n* = 6) and ISO 2w side (*n* = 6). Two‐way repeated‐measures ANOVA with Sidak's post hoc test was performed. *p < 0.05 CTL versus ISO 2w.

In Experiment 1, after 2 weeks NMES‐ISO loading, collected gastrocnemius muscles from ISO 2w group showed connective tissues covering the proximal side of the medial portion (Figure 2A). Wet weight of plantar flexor muscles and gastrocnemius muscles did not show a significant difference between CNT and ISO 2w groups (plantar flexor muscles: CNT 1388 ± 75.3 vs. ISO 2w 1442 ± 113.2 mg, *p* = 0.2899; gastrocnemius muscle: CNT 1095 ± 64.4 vs. ISO 2w 1143 ± 108.4 mg, *p *= 0.3291) (Figure 2B). In vivo torque analysis showed that maximal isometric torque normalized by body weight was significantly decreased on the ISO leg from 5th day to the last day of loading compared to CTL leg (Figure 2C). These results suggests that 2 weeks of continuous ISO do not impact muscle mass but impair muscle function.

### 3.2 Repeated Muscle Contraction Using N**MES** Causes Reversible Fibrogenic Lesion Within the Muscles

**Figure 3:**
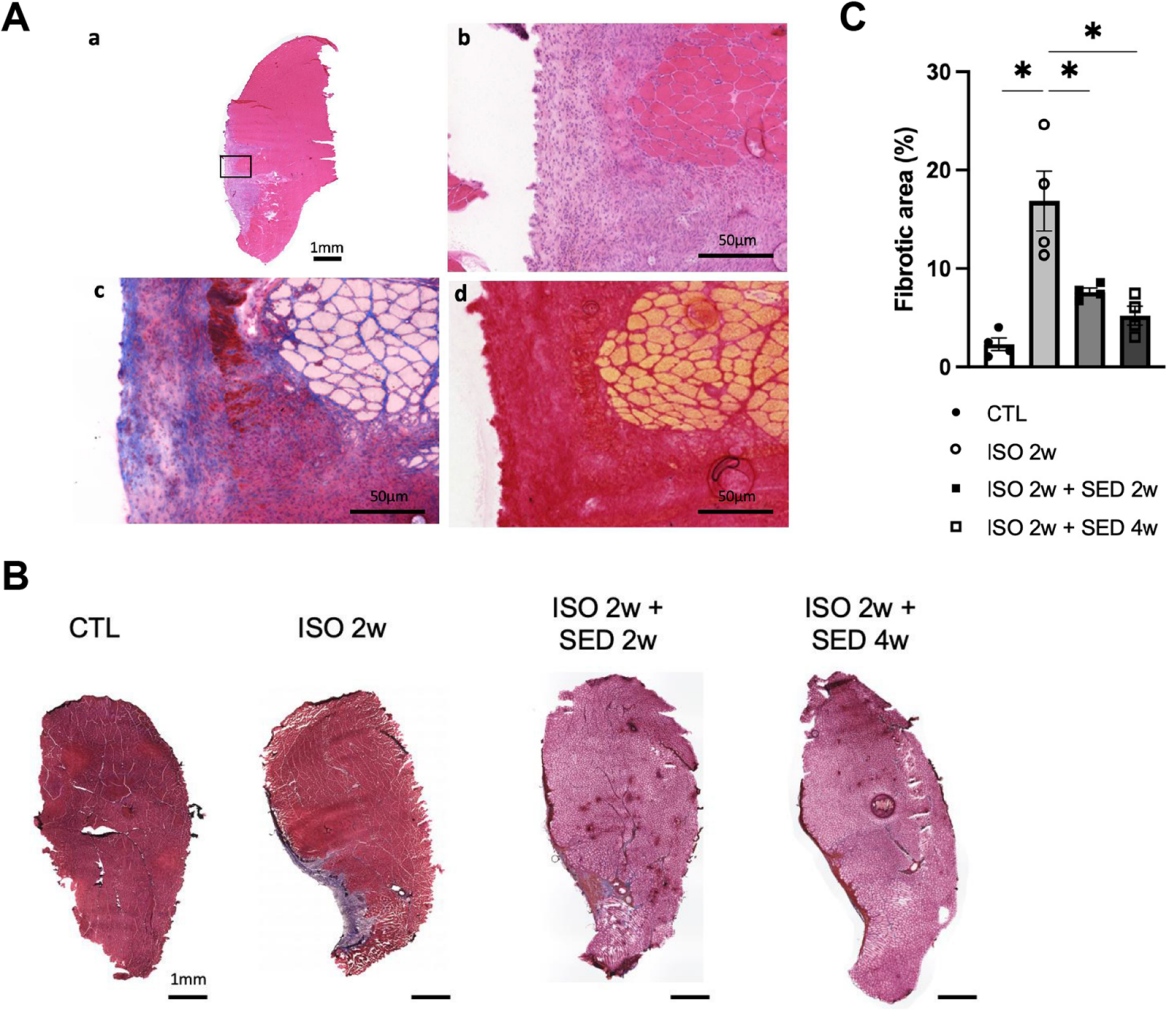
(A) Representative images of serial transverse sections of medial gastrocnemius muscles stained with hematoxylin and eosin (HE) (a and b). Masson Trichrome (MT) (c), and Sirius red (d) after two weeks of continuous ISO. Panels b, c, and d are enlarged views of the areas squared in panel a. Scale bars: 1 mm (a) and 50 µm (b, c, and d). (B) Representative images of transverse sections of medial gastrocnemius muscles stained with MT after two weeks of continuous ISO with or without sedentary periods. Scale bars = 1 mm. (C) Quantitative analysis of the fibrogenic area of the muscles from the CTL (*n* = 4), ISO 2w (*n* = 4), ISO 2w + SED 2w (*n* = 4), and ISO 2w + SED 4w (*n* = 4) groups. Data are means ± SD. *p < 0.05 with one‐way ANOVA followed by Tukey's post hoc test for comparison of groups.

Next, we conducted histological analysis to capture the phenomena occurring within the muscles after 2 weeks of continuous ISO. HE staining revealed that in the medial gastrocnemius muscle collected from ISO 2w group, areas with disrupted normal muscle fiber structure and numerous cells were focally distributed in the superficial region of the gastrocnemius muscle (Figure 3A). Interestingly, muscle fibers with centrally located nuclei, which indicate regenerating muscle fibers, were observed only in some fibers situated at the boundary between these lesions and the normal muscle fibers. Masson‐Trichrome staining and Sirius red staining revealed that this area contained collagen‐rich lesions. In addition, we compared the ISO 2w group with the ISO 2w + SED 2w and ISO 2w + SED 4w groups to determine whether the lesions were reversible. Masson‐Trichrome staining showed that gastrocnemius muscles in the ISO 2w group had a significant increase in the fibrogenic areas compared to those in the CTL group, while the gastrocnemius muscles in the ISO 2w + SED 2w group and the ISO 2w + SED 4w group showed a significant reduction in the fibrogenic areas compared to those in the ISO 2w group (Figure 3B,C).

### 3.3 Excessive Muscle Contraction Using NMES Induces Reversible Changes Detectable by MRI in the Proximal to Middle of the Muscle

**Figure 4:**
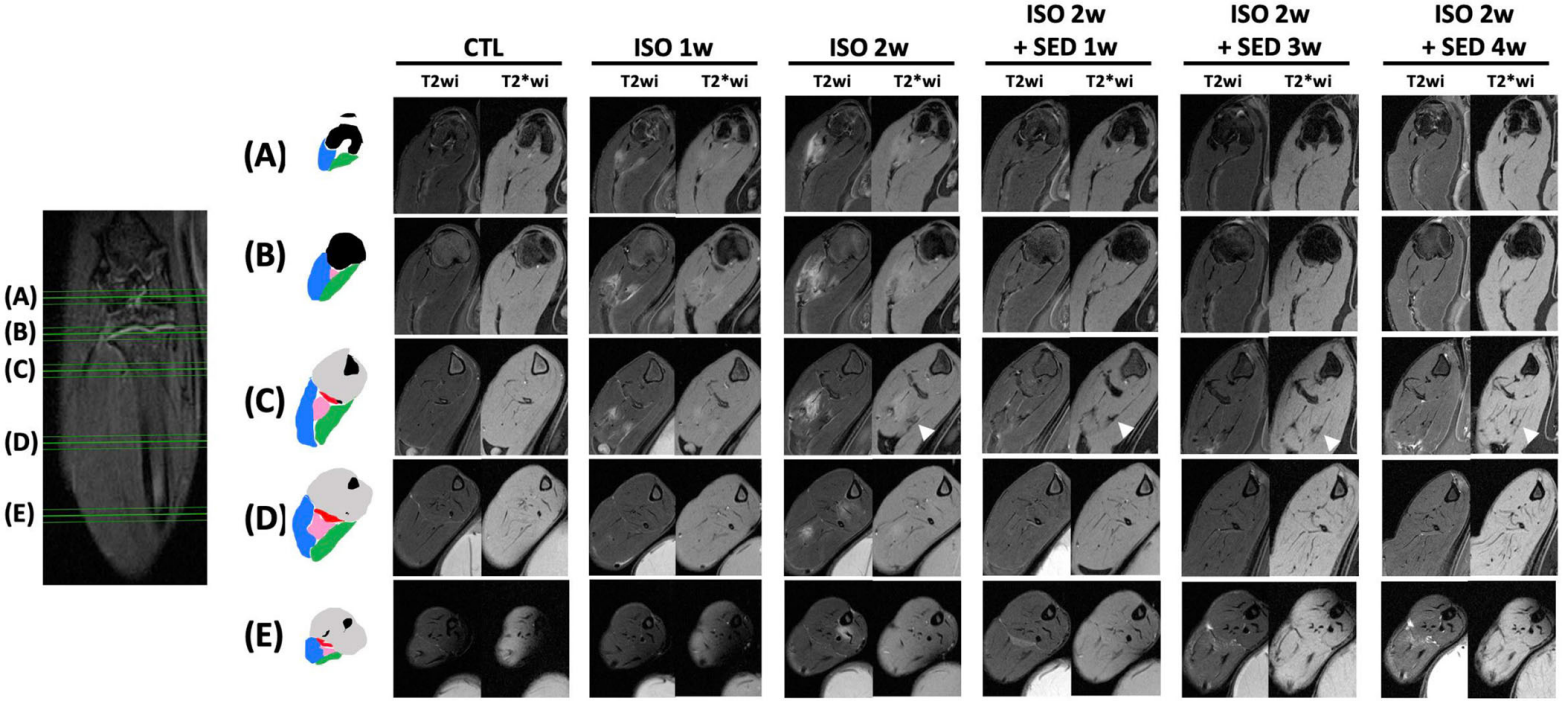
In vivo MRI analysis of the longitudinal course of skeletal muscle affected by continuous ISO. Regarding the levels shown in the representative T2wi of the coronal plane on the far left: the joint surface level (A), 3.4 mm distal (B), 6.8 mm distal (C), 13.6 mm distal (D), and 20.4 mm distal (E) to the joint surface level, representative T2wi and T2*wi of the axial plane of the right lower leg in CTL, ISO 1w, ISO 2w, ISO 2w + SED 1w, ISO 2w + SED 3w, and ISO 2w + SED 4w, as well as schematic diagrams of the structures within the image (black indicates bones, blue indicates the lateral gastrocnemius muscle, green indicates the medial gastrocnemius muscle, red indicates the soleus muscle, and pink indicates the plantaris muscle), are shown. Arrowheads in T2*wi indicate a low‐density area in the medial gastrocnemius muscle.

We further used MRI to determine the spatial location of the lesions caused by continuous ISO and to capture changes over time (Figure 4). After 1 week of continuous ISO, we observed a high intensity T2wi signal in the proximal to middle regions of the medial and lateral gastrocnemius muscles. After 2 weeks of continuous ISO, the high intensity T2wi signal increased in extent, and a low intensity T2 star signal appeared in the superficial layer of the medial gastrocnemius muscle. Following 2 weeks of continuous ISO and 1 week of sedentary, the high intensity area in T2wi disappeared, but the low intensity signal in T2*wi remained. After 2 weeks of continuous ISO and 3 weeks of sedentary, and subsequently 4 weeks of sedentary, the low intensity signal in T2*wi gradually diminished. When evaluating the fibrogenic areas in the proximal, middle, and distal thirds of the medial gastrocnemius muscle, the fibrogenic areas corresponded to the regions indicated by the high intensity MRI signals. (Supporting Information S1: Figure S1).

### 3.4 Excessive Muscle Contraction Using NMES‐Induced Intramuscular Fibrogenic Lesion Does Not Require the Disruption of Muscle Fibers

**Figure 5:**
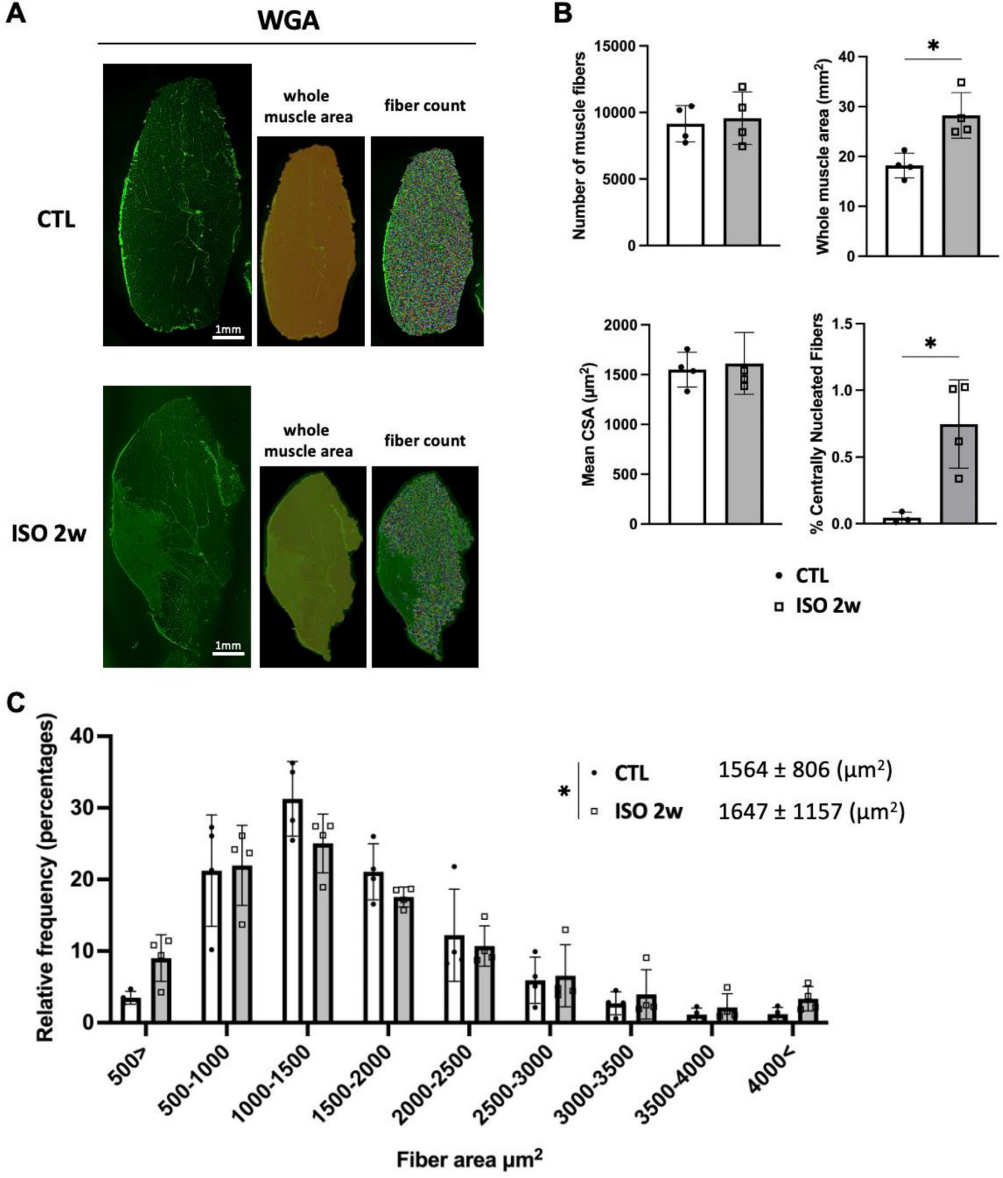
(A) Representative images show the WGA‐stained medial gastrocnemius muscle from CTL and ISO 2w groups and processed for analysis. The area covered with transparent yellow is the calculated whole muscle area, showing all areas covered by the epimysium. Randomly colored muscle fibers indicate the calculated number of muscle fibers and their area. (B) Quantitative analysis of the number of muscle fibers, mean CSA, whole muscle area, and the proportion of centrally nucleated fibers of the muscles from the CTL (*n* = 4) and ISO 2w (*n* = 4) groups. Data are means ± SD. *p < 0.05 with unpaired *t*‐test for comparison of groups. (C) The frequency histogram showing the distribution of muscle fiber area in gastrocnemius muscle from CTL (*n* = 4) and ISO 2w (*n* = 4) groups. *p < 0.05 with Kolmogorov–Smirnov test for comparison of frequency distributions of two groups.

To evaluate the effects of 2 weeks of continuous ISO on muscle fibers, we conducted a morphological analysis of the muscle fibers in the medial gastrocnemius muscle using immunohistochemistry. There was no significant difference in the number of muscle fibers and CSA between the CTL group and the ISO 2w group (number of muscle fibers: CNT 9152 ± 1373 vs. ISO 2w 9566 ± 1981, *p* = 0.7431; CSA: CNT 1551 ± 174.4 vs. ISO 2w 1613 ± 311.5 µm^2^, *p* = 0.7385). However, the whole muscle area was significantly increased in the ISO 2w group compared to the CTL group plantar flexor muscles (CNT 18.2 ± 2.5 vs. ISO 2w 28.2 ± 4.6 mm^2^, *p* = 0.0083). The proportion of centrally nucleated fibers among all muscle fibers was higher in the ISO 2w group than in the CTL group (CNT 0.04 ± 0.034 vs. ISO 2w 0.75 ± 0.332%, *p *= 0.0055) (Figure 5A,B), but the absolute value was less than 2%. The frequency distribution analysis indicated that muscle fibers in the ISO 2w group had a significantly larger diameter than those in the CTL group (CNT 1564 ± 806 vs. ISO 2w 1647 ± 1157 µm^2^, *p *< 0.0001). Additionally, the distribution of CSA fiber area showed a trend of an increased proportion of both smaller and larger fibers in the ISO 2w group compared to the CTL group (Figure 5C).

**Figure 6:**
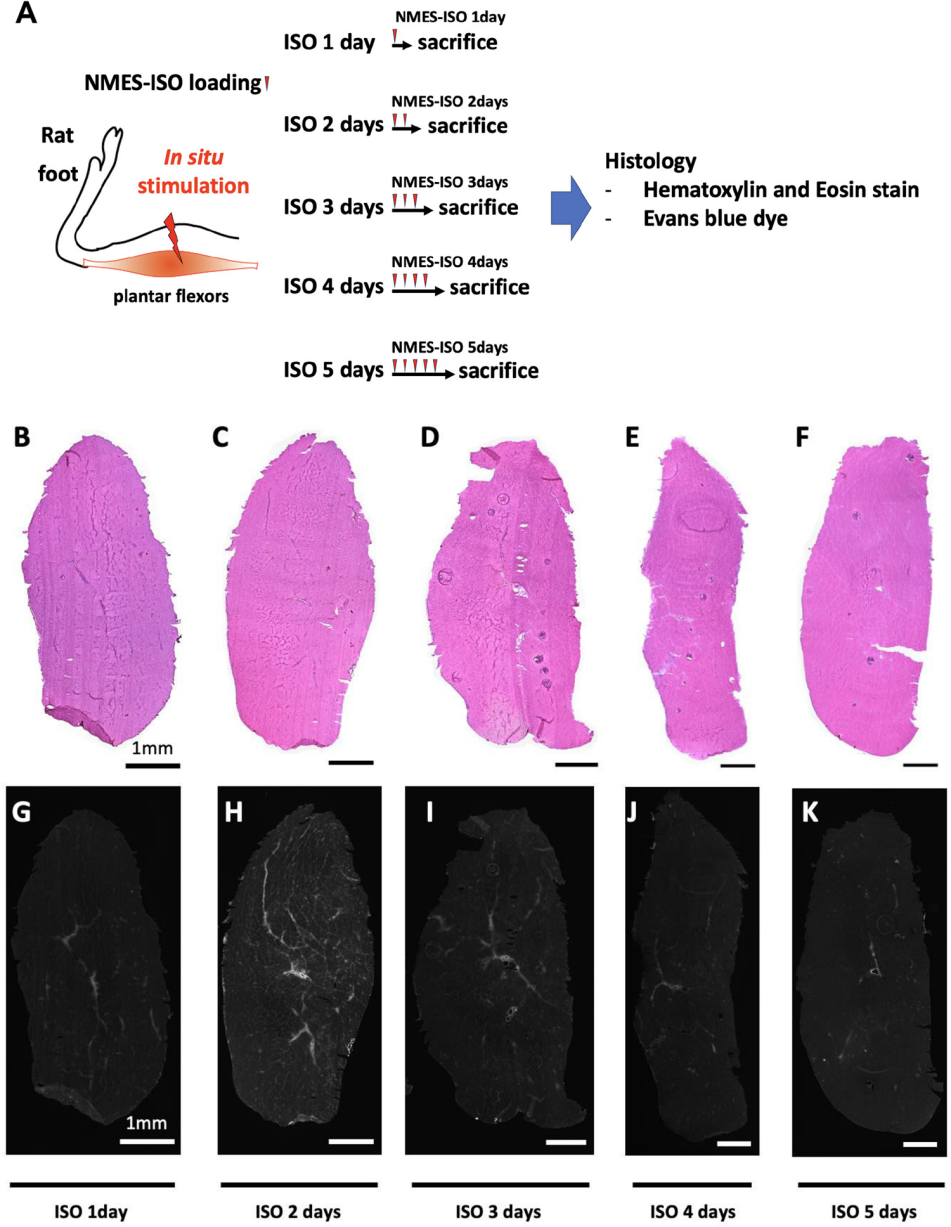
(A) Schematic overview of the Experiment 2. Representative images of serial transverse sections of medial gastrocnemius muscles stained for Haematoxylin and Eosin (HE) (B–F) and Evans Blue dye (EBD) (G–K) after ISO 1 day, ISO 2 days, ISO 3 days, ISO 4 days, or ISO 5 days. Scale bars 1 mm.

Next, we confirmed the histological changes induced by NMES‐ISO loading continuous stress from 1–5 days to verify whether muscle damage occurs in the early stages of exercise (Figure 6A). HE stains did not show structural disruption of muscle fibers in the medial gastrocnemius muscle of any rats given NMES‐ISO load for 1–5 days (Figure 6B–F). Only a very small number of EBD‐positive muscle fibers were detected in the medial gastrocnemius muscle of any rats given NMES‐ISO load for 1–5 days (Figure 6G–K). These results do not support the theory that the cascade of mechanisms, involving inflammatory cell recruitment and the induction of intramuscular fibrosis, arises from the disruption of muscle fibers due to overuse.

### 3.5 Excessive Loading of NMES‐ISO Promotes the Intramuscular Proliferation of FAPs

**Figure 7:**
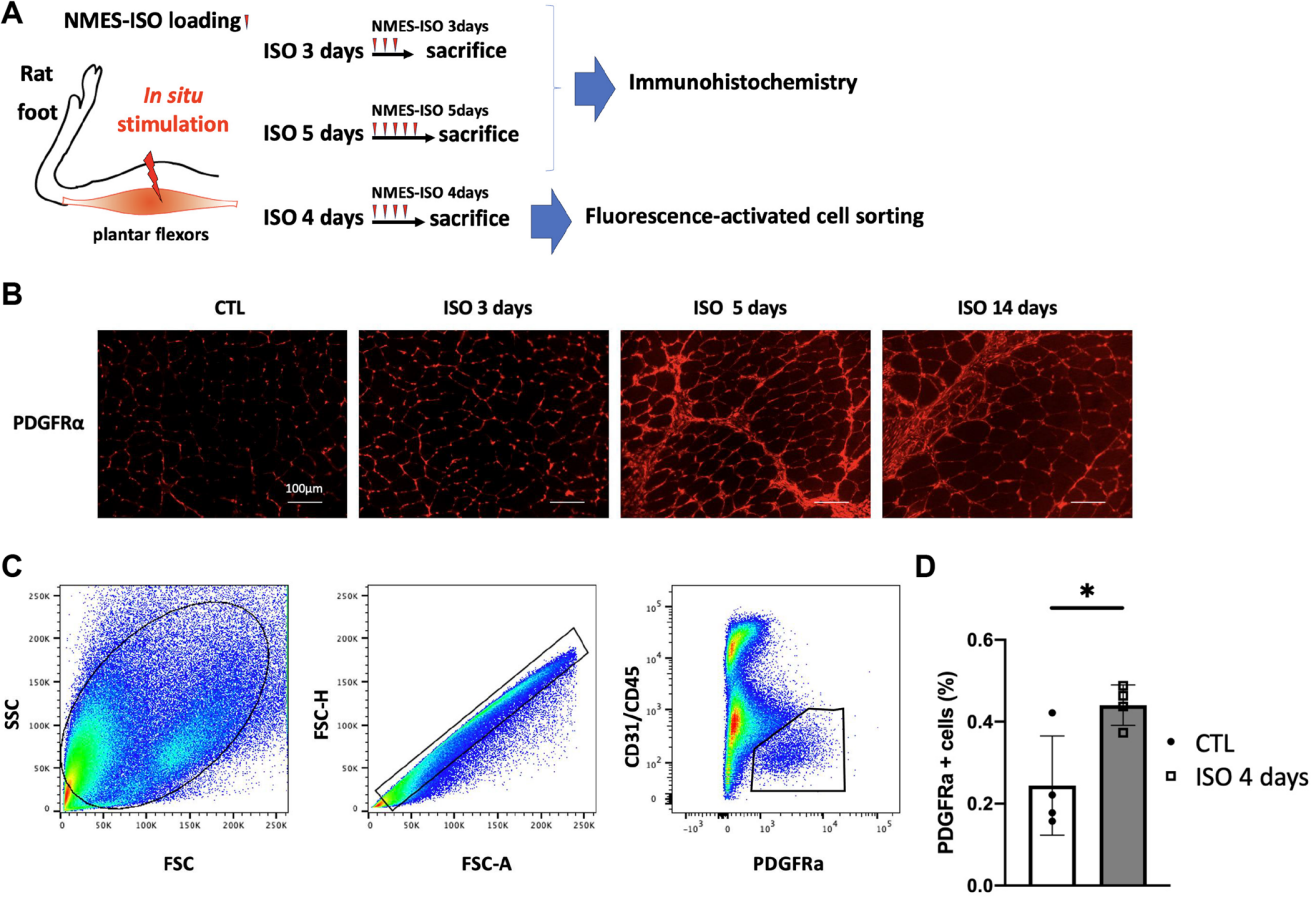
(A) Schematic overview of the Experiment 3. (B) Representative image immunofluorescence staining for PDGFRα in gastrocnemius muscles from CTL, ISO for 3 days, ISO for 5 days, and ISO for 14 days groups. (C) Gating strategies used in flow cytometry analysis. (D) Percentage of PDGFRα‐positive cells from gastrocnemius muscles of CTL (*n* = 4) and ISO for 4 days (*n* = 4) groups. *p < 0.05 with unpaired *t*‐test. Data shows mean ± SD.

In Experiment 3, we focused on the behavior of cells surrounding muscle fibers and evaluated the proliferation of FAPs using immunohistochemistry and flow cytometry (Figure 7A). The PDGFRα‐positive cells, a marker for FAPs, were found to have increased at Day 3 of NMES‐ISO loading (Figure 7B). Using the method established in mice [20], we confirmed that sorted PDGFRα‐positive cells in rats also proliferate and differentiate into adipocytes with medium treatment, and as a result, FAPs from rat skeletal muscle can be collected　(Supporting Information S1: Figure S2). To accurately quantify the proliferation of FAPs, we counted PDGFRα‐positive cells in the muscles at Day 4 of NMES‐ISO loading. There was a significant increase in the number of PDGFRα‐positive cells compared to the rats that did not receive the load (Figure 7C).

## 4 Discussion

Overuse is thought to cause primarily microdamage to muscle fibers, leading to the prolonged presence of inflammatory cells within the muscle, which disrupts the balance of the inflammatory response and results in muscle weakness and fibrosis [1]. However, our findings, obtained through detailed investigation of the effects of overuse using NMES, challenge this conventional understanding and suggest that repetitive, non‐damaging training may induce fibrosis, with the activation of FAPs as the inciting event in muscle.

Our previous study using NMES‐ISO with adequate rest periods demonstrated muscle hypertrophy effects [16], increased muscle endurance, and improved mitochondrial function [21]. However, as shown in this study, when NMES‐ISO is applied, particularly in a lengthened muscle position without sufficient recovery periods, muscle strength decreases and intramuscular fibrogenic lesions occur. We investigated intramuscular lesions using multiple stains. HE staining especially indicated that the lesions were not fully organized fibrotic regions, but rather clusters of cells resembling immature fibroblasts. Based on these findings, we infer that the intramuscular lesions observed in this study represent a stage before fully developed fibrosis. Supporting this, the fibrogenic regions showed improvement during the recovery period following cessation of the exercise load. The fibrogenic area observed in this model appears to be relatively acute, and given the presence of multiple cell types, it might be more appropriate to describe it as granulation tissue [2, 22].

In addition to the fibrogenic alterations, NMES‐ISO decreased muscle strength without any overt tissue damage. Several hypotheses have been proposed to explain muscle weakness associated with overuse, including the glycogen depletion hypothesis, muscle damage hypothesis, inflammation hypothesis, and oxidative stress hypothesis [3]. These factors are believed to contribute to a state of prolonged force depression, resulting in decreased strength without a corresponding loss in muscle mass, as observed in our results. In this regard, the model used in this study is considered valid for replicating OIMD. Recently, it has also been demonstrated that overuse using NMES leads to myofibrillar dysfunction [23]. While intramuscular fibrosis is clearly an important therapeutic target, these findings suggests that additional mechanisms contributing to impaired muscle function may exist and should be addressed in future research.

Noninvasive methods to assess fibrosis in tissues are still challenging in both preclinical and clinical settings. In this study, MRI proved useful for evaluating the temporal and spatial effects of excessive muscle contraction stress. T2*wi, which acquires images using gradient‐echo sequences with shorter repetition time and echo time, allowing faster image acquisition, has been suggested to have potential for detecting fibrosis within tissues [24, 25]. In musculoskeletal diseases, T2*wi is used to assess the continuity of tendon components [26] and is increasingly being applied clinically as a guideline to determine return‐to‐play timing following aponeurosis tears associated with muscle strains. Similarly, in this study, T2*wi was more effective than T2wi in identifying the location of fibrogenic regions and monitoring their resolution over time, highlighting its potential as a promising tool for evaluating muscle injury and recovery.

Based on the data from our current study, 2 weeks of continuous ISO did not result in either muscle atrophy or hypertrophy, but it did cause a focal fibrogenic lesion in the superficial layer of the medial gastrocnemius muscle. In previous studies on intramuscular fibrosis due to overuse, fibrotic changes were typically observed in highly magnified images of specific muscle regions, while fibrosis was generally described as being diffusely distributed within the interstitial spaces [5, 27]. We believe this difference is due to the method of exercise loading. Even if electrical stimulation uniformly recruits muscle fibers, it is physiologically unlikely that the same level of stress would be imposed on each fiber. Biomechanical studies have demonstrated that stress distribution within a single muscle can vary depending on factors such as fiber position, fiber length, and the surrounding environment [28]. In our study, NMES‐ISO was performed daily under anesthesia at the lengthened muscle position using the same settings and intensity, and as a result, regions within the muscle that are more susceptible to mechanical loading were likely subjected to especially high and repeated stress.

Moreover, when NMES‐ISO was performed at the shortened position, no fibrogenic lesions confined to the superficial layer were observed in the middle to distal regions of the muscle where the surface electrodes were applied (see Supporting Information S1: Figure S1). This suggests that direct induction of lesions by electrical stimulation itself via surface electrodes is not a plausible explanation. Consistent with this, in the current study, no skin injuries characteristic of electrical burns were observed (data not shown). Furthermore, our previous study using the same surface electrode configuration demonstrated that NMES‐ISO applied to the gastrocnemius muscle at the optimal length for 600 repetitions every other day over a 4‐week period resulted in minimal muscle damage in the middle region, where the electrodes were placed [18].

It is reasonable to consider that cytokines released from disrupted muscle fiber membranes due to exercise‐induced stress may continuously attract inflammatory cells [1]. Indeed, in DMD mice, which are prone to muscle fiber damage due to the absence of dystrophin, EBD‐positive muscle fibers are ubiquitously detected throughout the muscle even in a sedentary state [17], and intramuscular fibrosis increases progressively over time [29]. However, our findings revealed that, while a few regenerating muscle fibers were observed adjacent to the fibrogenic areas, no significant muscle damage was present even after five consecutive days of repeated muscle contraction loading. Additionally, the possibility that the fibrogenic lesion resulted from muscle fiber destruction and replacement or that widespread muscle fiber damage occurred between Day 5 and 2 weeks is inconsistent with the histological evidence showing no significant reduction in muscle fiber count and a markedly low presence of regenerating muscle fibers after 2 weeks of NMES‐ISO. These suggest that disruption of muscle fibers, which would typically result in sustained cytokine release, may not be essential for the development of intramuscular fibrosis induced by excessive muscle contraction.

There are multipotent cells, known as FAPs, located around skeletal muscle fibers. Under specific conditions, such as rotator cuff tear, FAPs have been implicated in muscle degeneration, including fibrosis [12, 30]. Furthermore, mechanical stimuli have been shown to trigger both the proliferation and differentiation of FAPs [31]. Considering prior research, the increase in FAPs observed in the present study suggests that these cells may have an enhanced propensity to differentiate into fibrogenic tissue in response to excessive exercise, thereby promoting fibrosis within the muscle.

There are several limitations in the present study. First, this model does not mimic the state of overuse involving repeated light labor as addressed in previous studies [5, 27]. However, overtraining occurs not only in endurance sports but also in strength training [32, 33, 34]. Against this background, we developed a model that closely resembles the load of resistance training. Second, we only examined the proliferation of FAPs. Our focus was primarily on demonstrating the characteristics of this model from a muscle physiology perspective. To gain a more detailed understanding of how FAPs respond to overuse stimuli, further research is needed. Third, due to the size constraints of the MRI device, relatively young rats were used. It is known that age significantly affects the characteristics of FAPs [35], and continuous NMES‐ISO in adult or elderly rats may produce different outcomes. Finally, given the advantages of using genetically modified species and the availability of abundant fluorescent immuno‐antibodies, mice offer many benefits. However, rats, in some instances, exhibit skeletal muscle phenotypes that more closely resemble those of humans [36]. The protocol for FAPs isolation from rat skeletal muscle reported in a previous study [37] is similar to ours, supporting the validation of studies targeting rat skeletal muscle.

## 5 Conclusions

This study establishes an OIMD model, characterized by muscle weakness and fibrogenesis, which may be useful for developing effective interventions for athletes and laborers who engage in excessive exercise without sufficient recovery. We propose that intramuscular FAPs may be involved early in the fibrogenic response to non‐damaging but excessive exercise stress.

## Author Contributions

Conceptualization: Hiroyori Fusagawa, Tatsuya Sato, Takashi Yamada, and Noritsugu Tohse. Methodology: Hiroyori Fusagawa, Akiyoshi Uezumi, Madoka Uezumi, Yuki Saito, Minami Fusagawa, Hiroyuki Takashima, and Takashi Yamada. Software: Hiroyori Fusagawa, Tatsuya Sato, and Takashi Yamada. Validation: Hiroyori Fusagawa, Tatsuya Sato, and Takashi Yamada. Formal analysis: Hiroyori Fusagawa, Tatsuya Sato, and Takashi Yamada. Investigation, Hiroyori Fusagawa, Azuma Naito, Nao Tokuda., and Nao Yamauchi. Resources: Hiroyori Fusagawa, Tatsuya Sato, Takashi Yamada, Nobutoshi Ichise, and Toshifumi Ogawa. Data curation: Hiroyori Fusagawa, Xuhui Liu, and Takashi Yamada. Writing – original draft preparation: Hiroyori Fusagawa. Writing – review and editing: Tatsuya Sato, Akiyoshi Uezumi, Yuki Saito, Hiroyuki Takashima, Brian Feeley, Xuhui Liu, and Takashi Yamada. Visualization: Hiroyori Fusagawa. Supervision: Tatsuya Sato, Nobutoshi Ichise, Toshifumi Ogawa, Takuro Karaushi, Noritsugu Tohse, Atsushi Teramoto, and Takashi Yamada. Project administration: Hiroyori Fusagawa, Tatsuya Sato, and Takashi Yamada.

## Conflicts of Interest

The authors declare no conflicts of interest.

## Supporting information


**Supplementary Figure S1:** Comparison between different types of two weeks NMES loadings. **Supplementary Figure S2:** Cell culture of sorted PDGFRα positive mesenchymal stromal cells.
