# Supplementary material for: Overuse‐Induced Muscle Disorder: Establishing a Rat Model to Unravel the Role of Fibro‐Adipogenic Progenitor Cells in Intramuscular Fibrosis
Source: J Orthop Res. 2025 Sep 30;43(12):2102–13. doi: 10.1002/jor.70076 (PMC12604459; doi:10.1002/jor.70076)
Supplement: Supplementary file 1 — Supplementary Figure S1: Comparison between different types of two weeks NMES loadings. Supplementary Figure S2: Cell culture of sorted PDGFRα positive mesenchymal stromal cells. [file JOR-43-2102-s001.docx]

**Supplemental data**

We examined the effects of continuous loading of NMES exercise with different contraction modalities. Mouse foot was secured in a foot plate at an angle of 40˚ dorsiflexion (i.e., 50˚ relative to the tibia: lengthened position of plantar flexor muscles) with NMES-ISO stimulation (0.5-ms monophasic rectangular pulse, 2-s contraction every 6 s. Each session consisted of four sets of five contractions at 5-min intervals and was carried out every day for 2 weeks) in ISO group. In the ECC group, the foot was secured in a foot plate at an angle of 0° dorsiflexion (i.e., 90° relative to the tibia). ECC involved forced dorsiflexion from 0° to 40°. Contractions of the plantar flexor muscles were induced by an electrical stimulator with 0.5-ms pulses at 100 Hz and 45 V for 2 seconds via surface electrodes. During the 2-second activation period, the ankle was forced by the stretching apparatus to undergo dorsiflexion of 40° to produce an ECC of the gastrocnemius muscles (angular velocity: 20°/s). This action was repeated every 6 seconds for 4 sets of 5 repetitions with a 5-minute interval between sets. The ISO-s group had the same stimulation protocol with a foot plate at an angle of - 40˚ plantarflexion (i.e., 130˚ relative to the tibia: shorten position of plantar flexor muscles). Masson-Trichrome staining of the medial gastrocnemius muscle after 2 weeks of daily treatment with each modality showed significantly increased fibrotic areas in the ECC group at the middle 1/3 level. For comparison purposes, data for the CTL and ISO groups were reused from the main text. (Supplementary Figure S1)


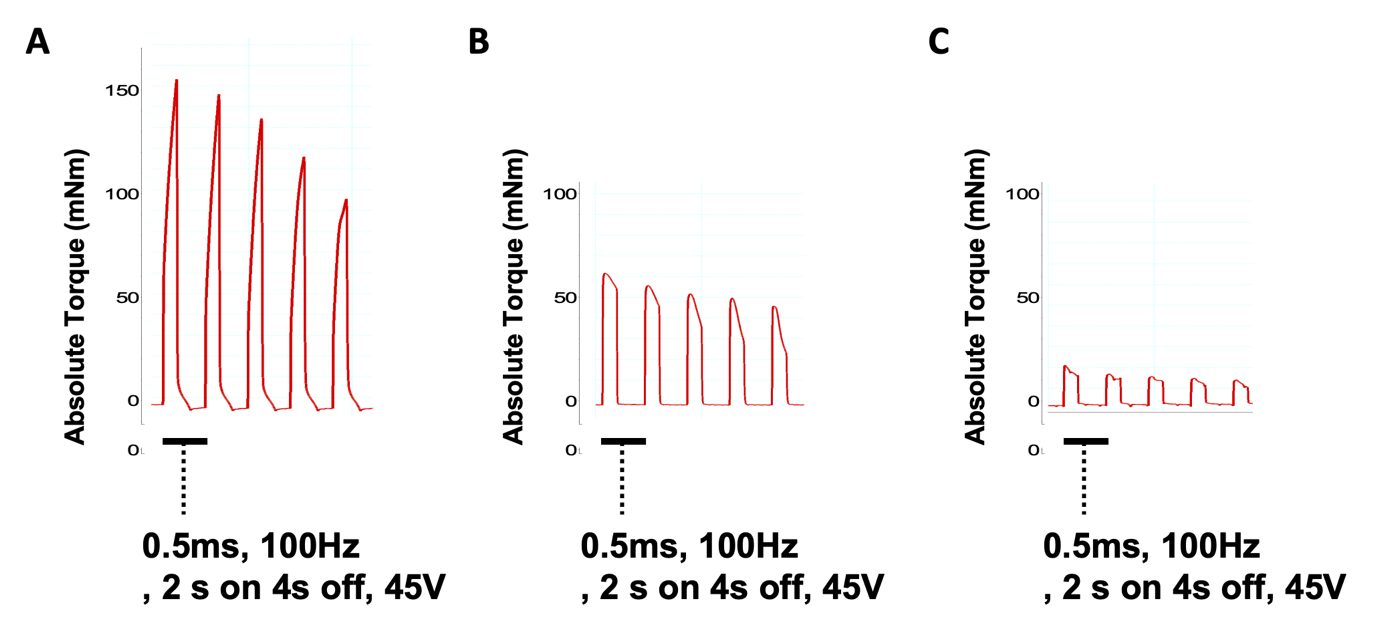


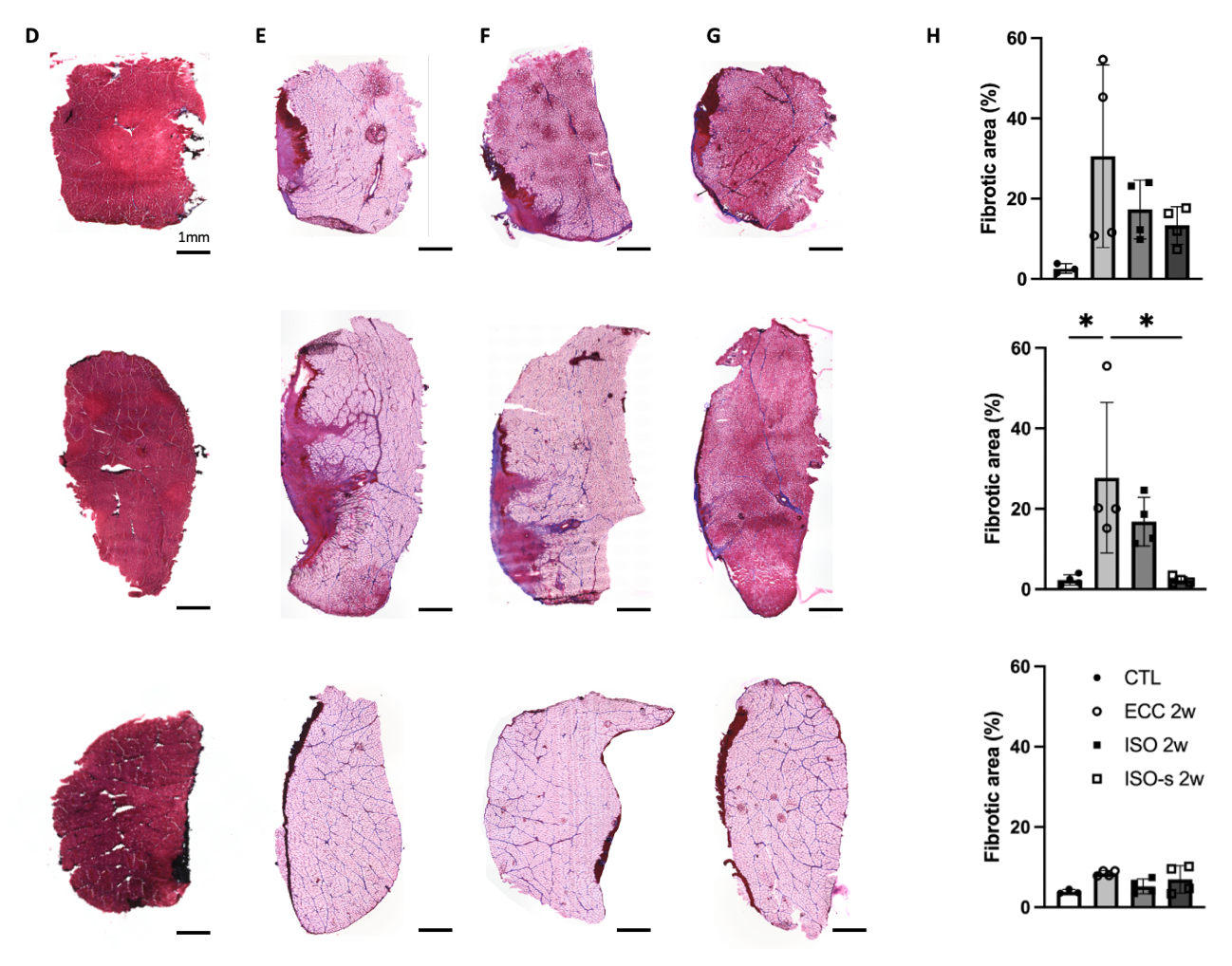


**Supplementary Figure S1. Comparison between different types of two weeks NMES loadings**

Typical torque traces of one set of NMES-induced eccentric contraction exercise (ECC) [4 sets of 5 contractions at 5-minute intervals, each consisting of a 2-second contraction at 100 Hz, 0.5 ms, and 45 V every 6 seconds, with forced foot dorsiflexion from 0° to 40° (angular velocity: 20°/s)] **(A)**, isometric contraction exercise (ISO) [4 sets of 5 contractions at 5-minute intervals, each consisting of a 2-second contraction at 100 Hz, 0.5 ms, and 45 V every 6 seconds, with the foot secured in a foot plate at an angle of 40° dorsiflexion] **(B)**, and  isometric contraction exercise with shortened plantar flexors (ISO-s) [4 sets of 5 contractions at 5-minute intervals, each consisting of a 2-second contraction at 100 Hz, 0.5 ms, and 45 V every 6 seconds, with the foot secured in a foot plate at an angle of 40° plantarflexion] **(C)**. Representative images of 1/3 proximal (upper panels), 1/3 middle (middle pannels), 1/3 distal (bottom panels) from CTL (**D)**, two weeks eccentric contraction loadings **(E)**, two weeks isometric contraction loadings **(F)**, two weeks isometric contraction loadings with shorten length of plantar flexors **(G)**. Quantitative analysis of the number of muscle fibers **(H)**. Data are means ± SD. *p < 0.05 with one‐way ANOVA followed by Tukey's post hoc test for comparison of groups.

We verified the accuracy of the digesting procedure and cell sorting, including antibodies for harvesting muscle MSCs from the plantar flexor muscle of rats. We sorted CD31 -/ CD45 -/ PDGFR⍺ + cells and seeded 3000 cells each in 98-well plates coated with Matrigel. The cells were cultured for 7 days in growth media (high glucose Dulbecco’s modified Eagle’s medium (DMEM) supplemented with 20% FBS, 2 mM GlutaMAX, and 1% penicillin-streptomycin). Subsequently, the cells were cultured for 4 days in adipogenic induction medium (DMEM supplemented with 10% FBS, 0.5 mM IBMX, 0.25 µM dexamethasone, 10 µg/ml insulin, and 1% penicillin-streptomycin), followed by 11 days in adipogenic maintenance medium (DMEM supplemented with 10% FBS, 10 µg/ml insulin, and 1% penicillin-streptomycin), resulting in evident adipogenic differentiation. (Supplementary Figure S2)


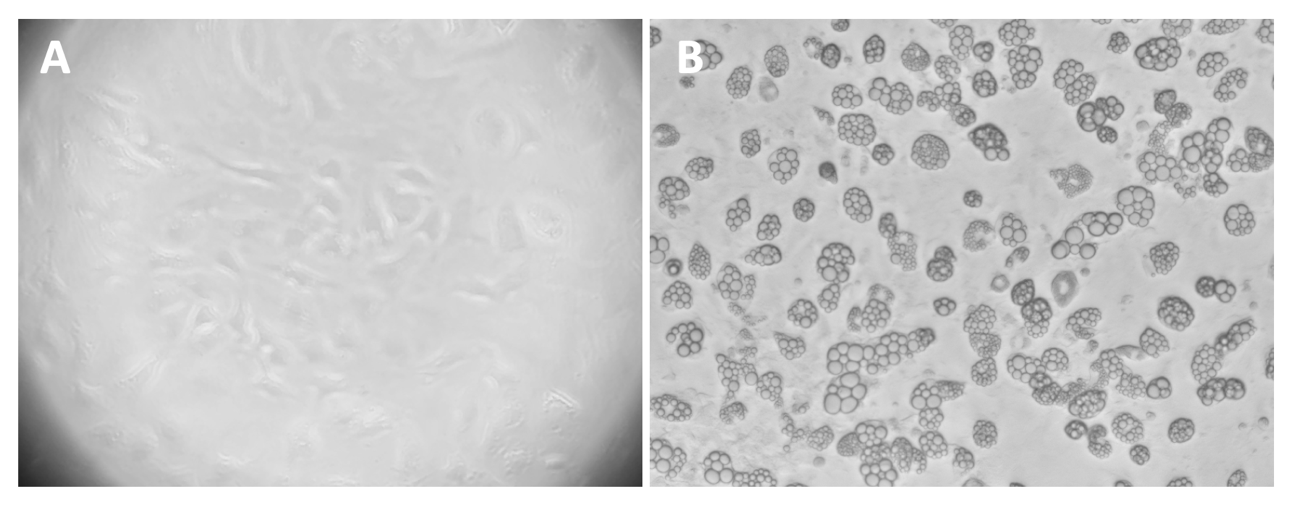


**Supplementary Figure S2. Cell culture of sorted PDGFRα positive mesenchymal stromal cells**

Morphology of cultured rat PDGFRα positive mesenchymal stromal cells in Growth media (**A**) and after adipogenic differentiation (**B**).
